# Comparison of Designated Coefficients and their Predictors in Functional Evaluation of Wheelchair Rugby Athletes

**DOI:** 10.1515/hukin-2015-0101

**Published:** 2015-01-12

**Authors:** Anna Zwierzchowska, Ewa Sadowska-Krępa, Marta Głowacz, Aleksandara Mostowik, Adam Maszczyk

**Affiliations:** 1 Department of Special Physical Education. The Jerzy Kukuczka Academy of Physical Education, Katowice, Poland; 2 Department of Physiological and Medical Sciences. The Jerzy Kukuczka Academy of Physical Education, Katowice, Poland; 3 Department of Sports Theory. The Jerzy Kukuczka Academy of Physical Education, Katowice, Poland

**Keywords:** wheelchair rugby, functional classification, regression models, correlation coefficients

## Abstract

The objectives of the present study were twofold: to determine differences between groups by means of chosen coefficients and to create significant predictors using regression models for athletes in wheelchair rugby who had the same spinal cord injury (tetraplegia) and were classified as low point and high point players. The study sample consisted of 24 subjects, who had sustained cervical spinal cord injury (CSCI). They were divided into low point (n=15) and high point (n=9) groups according to the IWRF Classification System. A one-way ANOVA revealed statistically significant differences in the following coefficients differentiating the groups: AC (η2=0.778), LC (η2=0.687), IC (η2=0.565), SC (η2=0.580). The Tukey’s HSD post-hoc test indicated statistically significant higher values of coefficients in the HP compared to the LP group: AC=0.958 (p=0.022), LC=0.989 (p=0.031), IC=0.971 (p=0.044), SC=0.938 (p=0.039). In the HP group, the most significant predictor was the sum of visceral and trunk fat which was negatively correlated with the SC (what constituted a positive adaptive change in response to training). With regard to the LP group, body height and circumference of the chest appeared to be most significant predictors and were positively correlated with the SC. In the LP group no predictor with respect to the SC was significantly correlated to sports training. Therefore, the functional classification system confirmed lower status of the LP players. The results of the present study indicate that both metabolic and somatic profiles which highly determine potential of wheelchair rugby athletes are significantly different in LP and HP players, what confirms the reliability of the functional classification system.

## Introduction

Sport and physical activity are widely accepted as necessary components of health. Over recent years, there has been increased emphasis on the role of sport and physical activity in enhancing health and quality of life of individuals with disability (Goldberg, 1995). Wheelchair rugby, as a contact game, is often recommended for individuals with paraplegia or tetraplegia. The game is characterized by frequent short-term efforts of maximum intensity and requires a high level of speed, strength and endurance. The complex pathophysiology of spinal cord injury (SCI) involves two mechanisms of injury: the primary one which is due to initial mechanical damage, and the secondary mechanism which is related to vascular, cellular and biochemical events. As a consequence of the primary mechanism of SCI, many organs, including these of the cardiovascular (CV) system, cease to be controlled by the autonomic nervous system (ANS) (Sadowska-Krępa et al., 2015). Response to physical activity fails to meet the needs of the body and typically results in low blood pressure (BP), what leads to reduced endurance and a decrease in sports performance. Autonomic dysreflexia (AD) occurs most often in individuals with SCI with spinal lesions above the T6 spinal cord level and can be voluntarily induced. Although AD may improve wheelchair racing performance in some athletes, it also elicits an increase in blood pressure, which can be dangerous. The International Paralympic Committee considers AD doping and has banned its use (Mills and Krassioukov, 2011).

Post-traumatic complications affect almost all body systems and may worsen the subject’s condition, as well as accelerate the progression of the disease. An enforced sedentary lifestyle and muscle paresis below location of injury are associated with adipose tissue accumulation in the trunk. Moreover, visceral fat accumulation favors the development of cardiac and metabolic diseases (Maruyama et al., 2008). Research indicates that high levels of visceral fat (VF) and trunk fat (TF) are highly correlated to the incidence of lifestyle-related diseases (Ford et al., 2013). Furthermore, the majority of studies on subjects with SCI have shown an unfavorable lipid profile caused by reduced serum high density lipoprotein cholesterol (HDL-C) and higher low density lipoprotein cholesterol (LDL-C) (Bauman and Spungen, 2008).

Classification is a unique and integral part of sport for people with disabilities. Its purpose is to ensure fair and equitable competition at all levels of sport and to allow athletes to compete at the highest possible level, despite individual differences in physical function. Functional classification systems ensure that athletes with a combination of impaired or absent upper and lower limb movement have an opportunity to practice a particular sports discipline and that the strategies and skills of competing teams and athletes, rather than the amount of movement of the athletes, are the factors determining success in competition. To be eligible to compete in wheelchair rugby, an athlete must have impairment disability caused by a verifiable and permanent health condition leading to activity limitation that impacts sports performance (McCann, 1987; Goldberg, 1995; ICIDH-2, 2000; IPC Classification Code, 2007). Wheelchair rugby (WR) is played by athletes with different levels of limitations of movement, strength and control in arms, the trunk and legs, allocated to one of the seven sport classes, ranging from 0.5 to 3.5 points.

The objectives of the present study were twofold: to determine differences between groups by means of chosen coefficients and to create most statistically significant predictors using regression models in wheelchair rugby athletes who had the same spinal cord injury (tetraplegia) and were classified as low point and high point players.

## Material and Methods

### Subjects

The study sample consisted of 24 men at the age of 34.2 ±5.2 years, who had sustained cervical spinal cord injury (CSCI) (Table 1). Mean training experience was 7 ±3.5 years. The subjects had been practicing wheelchair rugby once a week for 3 h for the past 5 to 14 years. They had sustained CSCI and suffered from paralysis of lower limbs and paresis of upper extremities (tetraplegia). The study participants were divided into *low point* (LP, n=15, sport classes 0.5–1.5) and *high point* (HP, n=9, sport classes 2.0 – 3.5) groups, according to the IWRF Classification System. None of the subjects had cardio-metabolic problems. All measurements were performed by the same qualified physiotherapist to minimize interobserver differences (Table 1).

### Procedures

The anthropometric measurements included body height (BH), waist (WC) and hip circumference (HC) (cm), as well as body mass (BM) (kg) (MENSOR Chair of EC type P3 150 K). Considering significant spasticity of subjects with CSI (foot dorsiflexion), body length was measured in the supine position with accuracy of the 0.01 m and the final result was an average of all 3 measurements. WC and HC were measured with an anthropometric tape over light clothing. Waist girth was measured at the minimum circumference between the iliac crest and the rib cage and hip girth at the maximum width over the greater trochanters.

Body fat content was determined with the use of the abdominal fat analyzer Viscan AB-140 (Tanita Corp, Tokyo, Japan) that utilizes bioelectrical impedance analysis (BIA). The Viscan apparatus has similar accuracy as DXA (gold standard). This new method of estimating abdominal fat by BIA represents a reliable tool for clinical evaluation of trunk fat (Zamrazilová et al., 2010).

The Tanita AB 140 is a novel device for indirect measurements of body fat in subjects who for various reasons cannot assume an upright position. The study was performed according to a standard protocol of the manufacturer. The AB140 is a device specifically designed for a wide range of needs from large scale research projects to routine clinical practice. It is convenient to work with disabled, critically ill and elderly subjects, moreover, duration of the non-invasive measurements is under 30 s. The device is easy to set up and use. Moreover, it gives highly accurate and repeatable results with minimal or no personal contact. Before the measurements are taken, the tested subject assumes a lying position for about 10 min. Hands are placed on the chest and the area to be tested is exposed. In subjects with severe spasticity, the lower limbs are stabilized by the person performing the test. The test should be performed after an overnight fast. This is the basis for calculating body composition by an algorithm which includes age, sex and body height (Kyle et al., 2004).

Following an overnight fast, blood samples of the participants were collected into heparinized tubes and immediately assayed for reduced glutathione using a colorimetric method according to Beutler et al. (1963). Samples of heparinized blood were centrifuged for 10 min at 1.000 g at 4°C to separate plasma and erythrocytes, that were then washed three times with cold (4°C) saline and kept frozen at −80°C for further analysis. Activities of superoxide dismutase (SOD), glutathione peroxidase (GPx), catalase (CAT) glutathione reductase (GR), concentrations of reduced gluthatione (GSH), uric acid, glucose, insulin, total cholesterol (TC), HDL-cholesterol (HDL-C) and triglycerides (TG) were assessed as previously described (Sadowska-Krępa et al., 2015).

Concentrations of LDL-cholesterol (LDL-C) were calculated using the Friedewald formula. To evaluate risk for vascular disease, the lipid ratios (TC/HDL-C, LDL-C/HDL-C, TG/HDL-C) and the atherogenic index of plasma [AIP=log10(TG/HDL) with TG and HDL-C expressed in molar concentrations were calculated (Dobiášová and Frohlich, 2001). The homeostasis model assessment (HOMA-IR) was used to estimate insulin resistance using the following formula:

$$\text{HOMA-IR}=\text{glucose}{\left(\text{mmol}/\text{L}\right)}^{*}\text{insulin}(\text{mlU}/\text{L})/22.5$$

The study was approved by the local Bioethical Committee of the Academy of Physical Education in Katowice, Poland (KB/15/2013). All tested subjects gave their written consent for participation in the study.

### Identification of coefficients

From the matrix of 28 variables, 4 coefficients were calculated. The lipid coefficient (LC) was estimated from 7 variables (TG, HDL, LDL, TC, TC/HDL, LDL/HDL and TG/HDL), the antioxidant coefficient (AC) from the next 6 (SOD, CAT, GPx, GR, GSH, KM), the insulin coefficient (IC) from 3 variables (HOMA-IR, insulin and glucose) and the somatic coefficient (SC) from 9 variables (BM, BH, hip circumference (HC), waist circumference (WaC), trunk fat (TF%), visceral fat (VF%), circumference of the chest (CC), circumference in full inhalation (CFI) and circumference in full exhalation (CFE)).

Indicating most significant predictors of the calculated coefficients is a complex task depending on modelling of frequently non-linear interactions (Van Den Tillaar and Ettema, 2009; Zehr, 2005). Non-linear tools are available to describe such phenomena (i.e. nonlinear regression and neural models), however, there is no agreement over the relative accuracy of such methods in predicting results (Maier et al., 2000; Maszczyk et al., 2011, 2012; Zehr, 2005).

### Statistical analysis

The data were analyzed using Statistica 9.1 software. Descriptive analysis consisted of the mean and standard deviation. For all measured variables, estimated sphericity was verified according to the Mauchly’s W test, and the Greenhouse–Geisser correction was used when necessary. Before using parametric tests, the hypotheses of normality and homogeneity of variance were verified using the Kolmogorov-Smirnov test and the Levene’s test, respectively. The comparison of analyzed values before and after the introduction of the experimental factor was carried out by means of analysis of variance ANOVA. When significant differences were found, Tukey HSD post-hoc tests were used. Classified according to Hopkins (2010), the effect size (eta-squared; η^2^) of each test was calculated for all analyses.

#### Regression Models

The multiple stepwise regressions were used to select the most statistically significant predictors (X_n_) of coefficients (Y_n_). In a nonlinear model, at least one derivative with respect to a parameter should involve that parameter. In this study, the Y_1_(t) = exp (a_1_t + b_1_t^2^) nonlinear regression model was used and verified after being transformed to a linear model using the transformation X_n1_(t) = ln Y_1_ (t).

The level of significance for all analyses was set at *p* ≤ 0.05.

## Results

Table 2 presents the values of coefficients in two groups HP and LP. A one-way ANOVA revealed statistically significant differences in the following coefficients differentiating the groups: AC (η2=0.778), LC (η2=0.687), IC (η2=0.565), SC (η2=0.580). The Tukey’s HSD post-hoc test indicated statistically significant higher values of coefficients in the HP compared to the LP group: AC=0.958 (p=0.022), LC=0.989 (p=0.031), IC=0.971 (p=0.044), SC=0.938 (p=0.039).

The most significant predictor for the lipid coefficient was TG/HD variable (Beta=0.947) in the LP group. For the antioxidant coefficient: GPx (Beta=0.467) and SOD (Beta=0.457); for the insulin coefficient: glucose (Beta=0.623); and for the somatic coefficient: CC (Beta=0.604) and BV (Beta=0.345). The regression models in the LP group had the following forms:

- Y1LP(AC)=0.944+0.016*GPx+0.017*SOD
- Y2LP(LC)=1.297–0.070*TG/HD
- Y3LP(SC)=1.066+0.004*CC-0.356*BV
- Y4LP(IC)=0.936–0.033*Glucose

In the HP group, the most significant predictors for the lipid coefficient were: TG (Beta=0.642), TC/HDL (Beta=0.231), LDL (Beta=0.220) and HDL (Beta=0.186). For the antioxidant coefficient: GSH (Beta=0.562), for the insulin coefficient: insulin (Beta=0.482) and HOMAR-IR (Beta=0.361). For the somatic coefficient: trunk fat% (Beta=0.875) and visceral fat% (Beta=0.716).

In the HP group the regression models had the following forms:

- Y5HP(AC)=0.960+0.011*GSH
- Y6HP(LC)=0.024+0.540*TG +0.215TC/HDL-0.128*LDL-0.115*HDL
- Y7HP(SC)=0.995-0.007*TF%+0.009* VF%
- Y8HP(IC)=0.869-0.027*Insuline-0.412*HOMAR-IR

## Discussion

In sports for people with disabilities that include numerous disciplines such as rugby, basketball, tennis, as well as track and field, functional classification systems have been introduced in order to ensure equitable competition considering particular disabilities. The functional classification systems allow selection of athletes with a similar level of functional capacity based on their ability to perform movements. Wheelchair rugby is a sports discipline for people with tetraplegia with different levels of movement limitation. Considering the level of disability, the IWRF (International Wheelchair Rugby Federation) has introduced the functional classification system that distinguishes between low point (LP, sport classes 0.5–1.5) and high point players (HP, sport classes 2.0–3.5). Consequently, scientists have searched for coefficients that will allow division of wheelchair rugby athletes into low point and high point players considering their different functional capacity. Based on somatic variables and the metabolic profile, the following coefficients were indicated: somatic (SC), lipid (LC), insulin (IC) and antioxidant (AC).

The calculated coefficients indicated significant differences in functional abilities between low point and high point athletes further confirmed by the differences observed in physiological training effects in subjects with tetraplegia. Although injury location was identical, somatic and physiological potential of wheelchair rugby players differed, what induced different physiological training effects in low point and high point players.

Previous studies have shown that long-term wheelchair rugby training elicits favorable changes in the lipid profile and antioxidant status (Sadowska-Krępa et al., 2015). However, there were no statistically significant differences between the evaluated variables in the LP and HP groups. In the present study, the antioxidant and lipid coefficients were found to be significantly higher in the HP group (Table 2).

It is well known that regular physical activity enhances mechanisms of antioxidant defenses by increasing activity of enzymatic and non-enzymatic antioxidants (Bloomer and Goldfarb, 2004; Gomez-Cabrera et al., 2008). Considering a high level of functional ability of HP players, better adaptation to exercise-induced oxidative stress is justified. The higher antioxidant capacity of the body, the more favorable changes in the lipid profile variables (an increase of HDL-C along with a concurrent decrease of LDL-C are observed).

In the present study, the greatest difference between the HP and LP groups was observed in the body’s response to oxidative stress (F – 25.035, p <0.022), although other variables were also statistically significant. Positive adaptive response to WR training is characterized by increased activity of red blood cell antioxidant enzymes (CAT and GPx) in both able-bodied and WR players, what has been recently confirmed in the study of Hübner-Woźniak et al. (2012). This is in line with the study of de Groot et al. (2003) in which it was indicated that physical activity in rehabilitation of subjects with SCI played an important role in the improvement of the lipid profile, insulin sensitivity and maintenance of daily living activities.

An enforced sedentary lifestyle and muscle paresis below location of injury are associated with adipose tissue accumulation in the trunk. Moreover, visceral fat accumulation is one of the factors increasing cardiometabolic risk (Maruyama et al., 2008; Siemińska, 2007). There are very few studies on this topic and most often they are not comparable as the experimental groups differ in terms of location of injury, time post injury and the level of physical activity (Gater, 2007; Gupta, 2006; Weil et al., 2002; Zwierzchowska et al., 2015).

The calculated regression models for the AC, LC, SC and IC in both LP and HP groups were quite diversified. However, it is worth noting that for Y7HP(SC) in the HP group, the most significant predictor was the sum of visceral and trunk fat which was negatively correlated with the SC (what constituted a positive adaptive change in response to training). With regard to the LP group, body height and circumference of the chest appeared to be most significant predictors and were positively correlated with the SC. Although changes of the SC resulting from training are not possible considering completion of the growth process. Thus, in the LP group, no predictor with respect to the SC could be indicated as correlated to sports training. Therefore, the functional classification system confirms lower status of LP players.

## Limitations of the study

The main limitations of this study were its cross-sectional design, a relatively small number of participants and lack of available literature on the subject to contrast the obtained data, what substantially limits the possibility of generalizing our findings.

The regression models confirmed the functional classification system in WR and its importance regardless of the injured segment of the spinal cord. Moreover, they can be reliable predictors for assessing training effects in athletes with SCI.

## Conclusions

The results of the present study indicate that both metabolic and somatic profiles which highly determine potential of wheelchair rugby athletes are significantly different in the LP and HP players, what confirms the reliability of the functional classification system. The predictors specific for particular coefficients and presented trends prove the necessity to diversify sports training in wheelchair rugby athletes with SCI in order to enhance their health condition.

## Figures and Tables

**Table 1:** Basic characteristics of the subjects

Variable	LP (n=15)	HP (n=9)
Age (years)	33.9 ±5.8	34.4 ±4.5
Age at time of injury (years)	16–20 (n=10) 21–25 (n=5)	18–20 (n=4) 21–25 (n=4) 27 (n=1)
Time post injury (years)	14.5 ± 5.5	13.8 ± 3.7
Level of spinal cord injury	C5 (n=9) C6 (n=2) C5–C6 (n=2) C6–C7 (n=2)	C7 (n=1) C5–C7 (n=4) C6–C7 (n=4)
AIS	B (n=9) C (n=5) D (n=1)	B (n=3) C (n=1) D (n=5)
Training experience (years)	6.8 ± 3.3	9.3 ± 4.3

Abbreviations: LP, low point WR players; HP, high point WR players; AIS, American spinal injury association impartment scale

**Table 2:** The differences between coefficients in the LP and HP groups with the results of the one-way ANOVA.

Variables	Group	Values	F	*p*
AC	I LP	0.936	25.035	0.022
	II HP	0.958		
LC	I LP	0.940	17.582	0.031
	II HP	0.989		
SC	I LP	0. 884	12.293	0.039
	II HP	0. 938		
IC	I LP	0. 933	10.008	0.044
	II HP	0. 971		
